# Appearance Comparisons, Affect, Body Dissatisfaction and Eating Pathology in Portuguese Female University Students

**DOI:** 10.3390/nu15112484

**Published:** 2023-05-26

**Authors:** Carol Coelho, Paulo Machado, Bárbara César Machado, Sónia Gonçalves

**Affiliations:** 1 Psychology School, University of Minho, 4704-553 Braga, Portugal; pg47097@alunos.uminho.pt (C.C.); pmachado@psi.uminho.pt (P.M.); 2 CEDH—Research Centre for Human Development, Faculdade de Educação e Psicologia, Universidade Católica Portuguesa, 4169-005 Porto, Portugal; bcmachado@ucp.pt

**Keywords:** appearance-based social comparisons, body dissatisfaction, eating disorders

## Abstract

Physical appearance comparisons have been theorized to be associated with negative indicators of body image. This study aimed to study appearance comparisons and their association to affect, body dissatisfaction, and eating pathology. Three hundred and ten female university students with ages between 17 and 25 years (M = 20.2, SD = 1.9) completed sociodemographic and clinical data, self-reported questionnaires, and questions about appearance comparisons. Among the participants, 98.71% reported making appearance comparisons, and 42.15% of these reported making them frequently or always. Higher reported frequencies of appearance comparisons were related to higher levels of body dissatisfaction, negative affect, and eating pathology. Appearance comparisons to acquaintances were the most frequent. Comparisons in person and through media were reported in similar proportions. Upward comparisons were more frequent than lateral and downward comparisons and were related to higher levels of body dissatisfaction than downward comparisons and to higher levels of body dissatisfaction, negative affect, and eating pathology than lateral comparisons. Upward comparisons to close peers were associated with higher body dissatisfaction as opposed to models/celebrities. Results, limitations, and implications are discussed.

## 1. Introduction

The Tripartite Influence Model [1] determines that there is pressure exerted by peers, family, and media to subscribe to appearance ideals that can originate in body dissatisfaction, which in turn may lead to eating pathology. This process would be mediated by the internalization of appearance ideals and by appearance-based social comparisons.

The concept of appearance comparisons was developed from Social Comparison Theory [2], which suggests that human beings have an innate necessity to evaluate their opinions and abilities and to seek to improve them, and that, in the absence of objective parameters, this evaluation is based on the comparison with others.

When appearance comparisons are made, the perceived discrepancy between the self and comparison target can elicit affective responses—such as anxiety, disappointment, envy, hope, or satisfaction. These responses can motivate behavioral responses such as involvement in invasive cosmetic surgeries [3] or weight-control maladaptive behaviors [4]. In their meta-analysis, Myers and Crowther [5] concluded that appearance comparisons are associated with body dissatisfaction and eating pathology.

Originally, Festinger [2] distinguished between two directions of comparison: upward—the target of the comparison is considered superior regarding the evaluated attribute—and downward—the target is considered inferior regarding the evaluated attribute. Taking that into account, Thornton and Arrowood [6] suggested that upward comparisons would be related to self-improvement and self-evaluation, while downward comparisons would be a form of self-enhancement, maintaining a positive self-image. Some studies (e.g., [7]) also used the concept of lateral comparisons—the target is evaluated as equal regarding the attribute.

Studies showed a higher frequency of upward appearance comparisons in relation to other directions [7,8,9] and a positive association between these and body dissatisfaction and negative affect [9]. Upward comparisons lead individuals to confront their own perceived inferiority [10]. Therefore, when realizing a discrepancy between the self and the comparison target, people may experience negative affect and feel motivated to engage in behaviors to diminish this discrepancy [2] (e.g., compensatory physical activity). Meanwhile, some studies [9,11] found that downward appearance comparisons were associated with higher levels of body satisfaction and positive affect and lower levels of negative affect. Nevertheless, other research verified that, independently of the comparison direction, appearance comparisons were associated with body dissatisfaction and eating pathology [12]. It is possible that, independently of its direction, appearance comparisons guide attention to appearance [4]. Another explanation for these results is that other factors can influence the consequences of these two types of comparison [13], such as the medium through which the comparison is made, the target of the comparison, or the perceived attainability of the target’s appearance—the degree to which the individual believes that is possible to achieve the appearance of the target.

Considering the complexity of the previous results, the overall goal of the current study was to determine the reported frequency of appearance comparisons and its direction, targets, and mediums. Furthermore, it sought to analyze the associations between these characteristics of appearance comparisons and body dissatisfaction, positive affect, negative affect, and eating pathology.

It was hypothesized that there would be significant associations between direction and target, direction and medium, perceived attainability and target, and perceived attainability and medium. Thus, comparisons to models were expected to be associated with a higher proportion of upward appearance comparisons and a lower perceived attainability [8]. Moreover, comparisons made through media were predicted to be associated with a lower perceived attainability and a higher proportion of upward appearance comparisons [7,8].

Higher reported frequencies of appearance comparisons were predicted to be associated with higher body dissatisfaction, negative affect and eating pathology, and lower positive affect [4]. Furthermore, upward appearance comparisons were predicted to be related to a higher body dissatisfaction, negative affect, and eating pathology and lower positive affect [9,14]. The previous association was hypothesized to be moderated by target [15] and medium, [7], but not BMI [16].

Upward appearance comparisons to models/celebrities were predicted to be associated with a higher body dissatisfaction than upward appearance comparisons to other targets [8,15], higher levels of negative affect and eating pathology, and lower levels of positive affect. The opposite associations were predicted for downward appearance comparisons to models/celebrities. The perceived attainability of the target’s appearance was expected to mediate the associations between target and psychological measures, within each direction of comparison [8].

## 2. Materials and Methods

### 2.1. Participants

The exclusion criteria were applied for age, gender, and nationality. Participants were 310 Portuguese female university students, with ages ranging between 17 and 25 years old (M = 20.2, SD = 1.9). The mean BMI was 22.7 (SD = 3.6), with 218 participants (70.3%) being integrated into the Normal BMI group, 25 (8%) Under the Normal BMI, and 67 (21.7%) Over the Normal BMI. Furthermore, 19 participants (6.1%) reported a diagnosis of an eating disorder: bulimia nervosa (*n* = 7), anorexia nervosa (*n* = 6), and binge eating disorder (*n* = 5). One of these participants did not identify the diagnosed disorder. Four participants were not included in the subsequent analyzes because they reported never making appearance comparisons.

### 2.2. Measures

Sociodemographic and Clinical Questionnaire: age, nationality, degree of qualification, occupation, questions about a diagnosis of an eating disorder, and a request for a participant’s code (composed of the first letter of the first and last name and the last three digits of the cell phone number).

Appearance Comparisons Questionnaire: participants were asked to indicate how often they make appearance comparisons with other women (never, rarely, occasionally, frequently, always). Next, they answered four questions adapted from Fardouly et al. [8]: (1) Who was your most recent appearance comparison with sister/cousin/close friend (i.e., close peer), acquaintance/distant friend/colleague (i.e., acquaintance), stranger, model/celebrity, N/A (in case you never make these comparisons), others (specify); (2) Through which medium the comparison was made: media (social media, internet, television, etc.), N/A (in case you never make these comparisons), others (specify); (3) How would you rate your appearance compared to this person: much worse, worse, the same, better, much better, N/A (in case you never make these comparisons); (4) How attainable/achievable is this person’s appearance: not at all, a little, very, very much, N/A (in case you never make these comparisons).

Positive And Negative Affect Schedule (PANAS; [17]; Portuguese version [18]) (20 items) measure the positive and negative affect associated with the reported appearance comparisons—“How did you feel in the most recent appearance comparison you made?”. Cronbach’s alphas were calculated for the full scale, α = 0.829, for the negative affect subscale, α = 0.919, and for the positive affect subscale, α = 0.838.

Body Image Subscale of the Body Investment Scale (BIS; [19], Portuguese version [20]) (6 items) established participants’ body dissatisfaction (e.g., “I am satisfied with my appearance”). Cronbach’s alpha obtained for the subscale was α = 0.912.

Eating Disorder Examination Questionnaire (EDE-Q; [21], Portuguese version [22]) (28 items) evaluated participants’ eating disorder symptoms in the past 28 days (e.g., “How many times has your weight influenced how you think (judge) yourself as a person?”), and participants’ weight and height. It is possible to calculate a global score and four subscales: restraint, eating concern, weight concern, and shape concern. The global score had a Cronbach’s alpha of α = 0.965, and the subscales of restraint, eating concern, weight concern, and shape concern resulted in alphas of 0.893, 0.847, 0.878, and 0.934, respectively.

### 2.3. Procedure

The study was conducted in accordance with the Declaration of Helsinki and has received the approval of the Ethics Committee for Research in Social and Human Sciences (CEICSH) of the University of Minho (CEICSH 127/2020).

Participants were recruited through the Credit System for Participation in Experiments of the School of Psychology of the University of Minho and through academic email. Before collaborating with the study, participants were informed about its confidentiality and about the possibility of abandoning it without any prejudice. The data collection took place over 2 months (October to December 2022), via Google Forms.

### 2.4. Data Analysis

An a priori power analysis was conducted using G*Power Version 3.1.9.6 [23,24] to determine the minimum sample size required to test the hypothesis of the study, using the software’s recommended values. For the Chi-square tests of independence, the minimum sample size needed was *N* = 232 (*df* = 6, effect size *f*^2^ = 0.3, *α* = 0.05, power = 0.95), while for the MANOVA tests, the minimum sample size needed was *N* = 184 (effect size *f*² = 0.0625, *α* = 0.05, power = 0.95, number of groups = 4, number of response variables = 8). Therefore, a sample size of 306 participants was suitable for the analyses conducted in the study.

Data processing was carried out using Microsoft Excel [25]. The answer to the question “How would you rate your appearance compared to this person” was used to code the direction of the appearance comparison: upward (much worse, worse), lateral (the same), and downward (better, much better). The BMI of the participants was calculated from the weight and height reported in the EDE-Q Questionnaire, and the variable Type of BMI was created (Below Normal < 18.5, Normal 18.5–24.9, Above Normal ≥ 25), based on the intervals indicated by the World Health Organization [26]. Afterward, the data were exported to IBM SPSS Statistics [27]. Normality analyses were conducted considering acceptable asymmetry values between −3 and +3 and kurtosis between −10 and +10, including standard error values [28]. All variables were within the recommended ranges, and there were no severe outliers, which allowed the inclusion of all participants and the use of parametric tests in the statistical analyses. Frequency, generalized linear model, Chi-square, and regression analyses were conducted on SPSS. Statistical significance was set at *p* < 0.05.

## 3. Results

### 3.1. Appearance Comparisons

Three hundred and six (98.71%) participants reported making appearance comparisons. Among these, 57 (18.63%) declared making appearance comparisons rarely, 120 (39.22%) occasionally, 102 (33.33%) frequently, and 27 (8.82%) always.

Appearance comparisons were reported in the following directions: 265 (86.6%) upward, 29 (9.48%) lateral, and 12 (3.92%) downward. One hundred and fifty-four (50.33%) appearance comparisons were made in person and 152 (49.67%) through media. The comparison targets specified were 96 (31.37%) acquaintances (acquaintance/distant friend/colleague), 90 (29.41%) models/celebrities, 73 (23.86%) strangers, and 47 (15.36%) close peers (sister/cousin/close friend).

Chi-square tests of independence were performed between the variables’ direction and target (not significant), target and perceived attainability of the target’s appearance (not significant), medium and perceived attainability of the target’s appearance (not significant), and direction and medium (*X*^2^(2, *N* = 306) = 5.13, *p* = 0.04). The contingency table between direction and medium indicated that 52.1% of the upward appearance comparisons occurred through media as opposed to 47.9% done in person. Furthermore, among the appearance comparisons made through the media, 90.8% were upward comparisons, while among the appearance comparisons made in person, 82.5% were upward comparisons.

### 3.2. Associations between Appearance Comparisons and Body Dissatisfaction, Positive Affect, Negative Affect, and Eating Pathology

The association between the reported frequency of appearance comparisons and body dissatisfaction, positive affect, negative affect, eating pathology, and EDE-Q subscales were analyzed using the generalized linear model. The reported frequency of appearance comparisons was associated with body dissatisfaction, *F*(3, 302) = 75.15, *p* < 0.001, negative affect, *F*(3, 302) = 46.09, *p* < 0.001, and eating pathology, *F*(3, 302) = 62.23, *p* < 0.001. More specifically, the reported frequency of appearance comparisons was associated with all EDE-Q subscales: restraint, F(3, 302) = 21.92, *p* < 0.001, eating concern, F(3, 302) = 37.74, *p* < 0.001, weight concern, F(3, 302) = 97.13, *p* < 0.001, and shape concern, F(3, 302) = 90.60, *p* < 0.001. Table 1 contains a post-hoc test with Bonferroni correction, which compares the observed means of the quantitative variables between the different levels of reported frequency of appearance comparisons. Thus, the greater the reported frequency of appearance comparisons, the greater the body dissatisfaction and shape concern. Moreover, the greater the reported frequency of appearance comparisons—except for the comparison between the reported frequencies rarely and occasionally—the greater the negative affect, eating pathology, restraint, weight concern, and eating concern.

To study the association between the appearance comparison direction and body dissatisfaction, positive affect, negative affect, eating pathology, and EDE-Q subscales, an analysis was performed using the generalized linear model. Thus, the appearance comparison direction was associated with body dissatisfaction, *F*(2, 303) = 19.40, *p* < 0.001, negative affect, *F*(2, 303) = 579.71, *p* < 0.001, and eating pathology *F*(2, 303) = 4.93, *p* = 0.008. Regarding EDE-Q subscales, the appearance comparison direction was associated with weight concern, F(2, 303) = 5.56, *p* = 0.004, and shape concern, F(2, 303) = 8.13, *p* < 0.001. Table 2 presents a post-hoc test with Bonferroni correction that compares the observed means of the quantitative variables between the levels of direction. This table indicates higher body dissatisfaction, negative affect, eating pathology, weight concern, and shape concern in upward appearance comparisons as opposed to lateral appearance comparisons and higher levels of body dissatisfaction in upward appearance comparisons as opposed to downward appearance comparisons.

The generalized linear model was used to analyze the moderation of the association between direction and body dissatisfaction, positive affect, negative affect, eating pathology, and EDE-Q subscales, with potential moderators being target, medium, and BMI. No significant moderation effects were found.

The data were divided in terms of direction to verify, through the generalized linear model, the association between the target and body dissatisfaction, positive affect, negative affect, eating pathology, and EDE-Q subscales, within each comparison direction. In the lateral and downward appearance comparisons, the target was not associated with these variables, while in the upward appearance comparisons, the target was associated with body dissatisfaction, *F*(3, 261) = 3.93, *p* = 0.009. Table 3 shows a post-hoc test with Bonferroni correction that compares the observed means of body dissatisfaction between the target levels, in upward appearance comparisons. It is concluded that there is greater body dissatisfaction in upward appearance comparisons to close peers as opposed to models/celebrities.

A linear regression verified that the association condition between the dependent variable (body dissatisfaction) and mediator (perceived attainability of the target’s appearance) was not fulfilled within the upward appearance comparisons. Thus, there is no mediation by perceived attainability of the target’s appearance of the association between the target and body dissatisfaction in the upward appearance comparisons.

## 4. Discussion

This study aimed to analyze university students’ appearance comparisons and their association with body dissatisfaction, positive affect, negative affect, and eating pathology.

The act of making appearance comparisons was prevalent amongst participants. Furthermore, of those who reported making these comparisons, a large number (42.15%) reported doing it frequently or always. These data corroborate the pervasiveness of appearance comparisons that have been previously reported [5]. According to Want [29], these comparisons are made automatically and not deliberately. The frequent occurrence of appearance comparisons can be explained from an evolutionary perspective. Thus, Gilbert et al. [30] propose that social comparisons may have been psychological mechanisms that served intrasexual selection, reciprocal altruism, parental investment, and the need for membership in specific social groups. The high frequency of appearance comparisons can be also understood according to the Tripartite Influence Model [1]. That is, the constant pressure exerted by society for the adoption of appearance ideals motivates frequent engagement in appearance comparisons, as they allow self-assessment of appearance and a consequent adjustment to accommodate these ideals.

Upward appearance comparisons were reported in greater numbers than appearance comparisons in other directions, consistent with the results found by Fardouly et al. [7,8] and Leahey et al. [9]. This result can be explained by Festinger’s [2] hypothesis that human beings have a unidirectional drive upward, that is, a tendency to make comparisons with others that are considered superior to improve abilities and opinions. Furthermore, according to Strahan et al. [31], women tend to be self-deprecating when evaluating their bodies, often selecting targets considered more attractive. In this way, it is possible that the inclination towards self-enhancement that exists in other types of comparison is countered in appearance comparisons by the message conveyed to women that their physical appearance will be judged according to cultural standards and will never live up to them.

While Fardouly et al. [7,8] verified a higher proportion of appearance comparisons made in person, in this study, there were similar proportions of appearance comparisons made in person and through media. This may be due to the increase in “screen time” related to the COVID-19 pandemic, as identified by Hu et al. [32] and Pišot et al. [33]. In this context, social distancing guidelines have encouraged greater use of social media as a form of communication and interpersonal interaction [34]. The increased use of highly visual social media, such as TikTok and Instagram, may explain the greater amount of appearance comparisons carried out through the media, as these social media platforms contain several photos and videos that contain potential appearance comparison targets—friends, family members, celebrities, among others. Indeed, Jiang and Ngien [35] found that a more frequent use of Instagram was associated with higher levels of social comparison.

Appearance comparisons to acquaintances were the most frequent, followed by models/celebrities, strangers, and close peers. By considering acquaintances, strangers, and close peers as similar targets and models/celebrities as dissimilar targets, these data are in line with those of Leahey and Crowther [15], who observed a higher frequency of appearance comparisons to similar targets than dissimilar ones. These results are also in line with Festinger’s [2] corollary that there is a tendency to make comparisons with targets with similar abilities. On the other hand, the number of appearance comparisons with models/celebrities was very similar to the number of appearance comparisons with acquaintances. Although models/celebrities have abilities that are considered distant, they may not be dismissed as targets due to reflecting the cultural ideals of attractiveness by which participants believe they will be judged [36].

No significant association was found between direction and target, possibly due to the reduced number of lateral (29) and downward (12) appearance comparisons, a limitation also identified by other authors [7,8]. There were also no significant associations between the target and the perceived attainability of the target’s appearance and between the medium and the perceived attainability of the target’s appearance. A potential explanation for this is that the participants may not have been able to understand the concept of attainability, considering it in general terms other than personal ones. Moreover, although Fardouly et al. [8] found that models had an appearance considered less attainable than that of other targets, in this study, the participants seem to have considered the appearance of models/celebrities as attainable as that of the other targets. A possible interpretation of this is that models/celebrities are close to appearance standards, which can be misjudged in terms of attainability, given that the cultural norm often implies that appearance is highly controllable [31]. Another alternative explanation is that, currently, there is a greater diversity of appearances among models and celebrities, whether due to the growth of celebrities associated with more accessible media (such as YouTube) or due to society’s demand for greater inclusion in fashion, television, music, and others. According to Hunt and Ramón [37], there is a constant increase in the diversity of casts in television series. This greater diversity may provide closer targets, that is, more attainable, in terms of appearance.

A significant association was found between direction and medium. In agreement with this study’s hypothesis and the findings of Fardouly et al. [7,8], there was a greater proportion of upward appearance comparisons through the media than in person, and appearance comparisons made through the media had a higher proportion of upward comparisons than in-person comparisons. These data are not unexpected, because the images present in the media (television, magazines, social networks, etc.) are often manipulated [38], in addition to including targets that are usually closer to the appearance ideals—such as models. Furthermore, upward appearance comparisons may have been more frequent in media comparisons than in person due to some aspects of social media—such as likes and comments [7]—which make comparison information more salient. Moreover, other features of social networks—such as filters—can “beautify” photos and videos of potential targets, making upward comparisons more frequent.

The reported frequency of appearance comparisons was associated with body dissatisfaction, negative affect, and eating pathology, but not with positive affect. This may have resulted from the fact that some PANAS words are inappropriate to assess positive affect related to appearance comparisons, as defended by Fardouly et al. [8], who made changes to some words of the instrument.

We also concluded that the higher the reported frequency of appearance comparisons, the greater the body dissatisfaction of the participants. Assuming that the reported frequency of appearance comparisons is close to the real frequency with which participants make these comparisons, this finding is in line with Taniguchi and Ebesu Hubbard [4], who found that a higher frequency of appearance comparisons is positively related to body dissatisfaction. The same was verified by these authors regarding negative affect and eating pathology. In the present study, these measures were positively associated with the reported frequency of appearance comparisons, except for the comparison between the reported frequencies rarely and occasionally. Thus, the perception of making appearance comparisons with a low (occasionally) or very low (rarely) frequency does not seem to have implications in terms of negative affect and eating pathology.

Significant associations were obtained between appearance comparison direction and body dissatisfaction, negative affect, and eating pathology, but not between direction and positive affect, perhaps for the reason previously discussed regarding the PANAS. Moreover, the direction of comparison was associated with weight and shape concern, but not restraint and eating concern. This may have happened because the direction of comparison is more closely linked to constructs related to body image perception and evaluation than to constructs related to eating behavior and dieting.

Upward appearance comparisons, as opposed to lateral appearance comparisons, were linked to higher body dissatisfaction (as in [14]), negative affect, and eating pathology. Moreover, and in accordance with the results found by Fuller-Tyszkiewicz et al. [14] and Leahey et al. [9], higher levels of body dissatisfaction were found in upward appearance comparisons as opposed to downward appearance comparisons. These data can be justified by Wood’s [10] theory that upward comparisons can be demoralizing once the individual is forced to face his own inferiority.

We did not find differences in the levels of body dissatisfaction, negative affect, and eating pathology between lateral and downward appearance comparisons, and in the levels of negative affect and eating pathology between upward and downward appearance comparisons. The reduced number of downward appearance comparisons may justify these results.

As expected, and according to the results of Faith et al. [16], BMI did not moderate the association between direction and body dissatisfaction, positive affect, negative affect, and eating pathology. The pervasiveness of appearance comparisons [5] may explain why the different values of BMI did not influence the studied associations.

Contrary to expectations, there was also no moderation by target and medium. Since the evidence points to a significant association between these variables and direction [7,15], it is again hypothesized that the low number of downward and lateral appearance comparisons may explain the results.

In lateral and downward appearance comparisons, the target was not associated with body dissatisfaction, positive affect, negative affect, and eating pathology, and there was no mediation by perceived attainability of the target’s appearance. Similarly, Fardouly et al. [8] found no significant differences in levels of satisfaction with appearance, affect, and thoughts about diet and exercise when lateral and downward appearance comparisons were made to different targets. It is possible that the present study had the same limitation as Fardouly et al. [8], namely the reduced number of lateral and downward appearance comparisons to each target. 

In upward appearance comparisons, the target was not linked to positive affect, negative affect, and eating pathology, and there was no mediation by perceived attainability of the target’s appearance, similar to Fardouly et al. [8]. The authors proposed that the target’s appearance may be less relevant to affect and thoughts about diet and exercise than to satisfaction with appearance.

According to Wheeler [39], when making upward comparisons with similar targets, individuals may believe that they can improve their appearance, feeling motivated. In line with this idea, Leahey and Crowther [15] concluded that upward appearance comparisons with dissimilar targets are associated with greater body dissatisfaction than upward appearance comparisons with similar targets. In agreement, Fardouly et al. [8] observed higher dissatisfaction with appearance in upward appearance comparisons with models/celebrities (dissimilar targets) in opposition to other targets (similar), with mediation by perceived attainability of the target’s appearance, lower in relation to the appearance of models/celebrities. Myers and Crowther [5] found insignificant differences in levels of body satisfaction between appearance comparisons to peers (similar targets) and media images (dissimilar targets). Contrary to these findings, in this investigation, higher body dissatisfaction was found in upward appearance comparisons to close peers (similar targets) as opposed to upward appearance comparisons to models/celebrities (dissimilar targets), with no significant differences in levels of perceived attainability between the targets, and therefore unmediated by perceived attainability of the target’s appearance.

Indeed, Cash et al. [40] demonstrated that self-evaluation is more negative in upward appearance comparisons with targets presented as similar than with targets presented as professional models. These authors proposed that targets presented as similar would be considered a more appropriate standard of comparison than professional models. Similarly, Wood [10] states that upward comparisons can hurt self-esteem when the target judged superior is close or similar in the assessed dimensions.

Furthermore, according to Strahan et al. [31], when cultural norms are not salient, appearance comparisons with peers are more relevant than with models, causing greater discomfort. Thus, it is possible that cultural norms were less salient for the participants, leading them to devalue the appearance of models/celebrities, due to being aware that models/celebrities have more resources [8] and have a constantly manipulated image [38]. These results agree with Hoffman et al.’s [41] understanding that individuals are motivated by a concern with their comparability to other group members, excluding those perceived as definitely superior or inferior, and focusing on those perceived as being within the same range of ability.

Moreover, the comparison with close peers considered more attractive may not have increased the participants’ motivation, as proposed by Wheeler [39], but rather elicited feelings of guilt and, consequently, higher body dissatisfaction. The feeling of guilt can come from feeling impotence—“I could look like this, but I cannot”—or else from the fact that comparisons with closest people are perceived as an act of hostility towards them—“I shouldn’t be comparing myself to my sister”. Another factor to consider is that comparisons with close peers can include other comparison characteristics in addition to appearance, making the comparison even more threatening—“In addition to being prettier than me, she is also smarter”.

### 4.1. Limitations and Recommendations for Future Studies

The participants answered self-report questionnaires, so it is possible that some answers were influenced by failures in the interpretation of the questions or the tendency to give socially acceptable answers. According to Myers and Crowther [5], self-report questionnaires may have biased responses, however, they allow direct access to the social comparison process. Despite these limitations, Leahey et al. [9] argue that self-report questionnaires may be the most appropriate and accurate way to obtain data on affect, cognitions, and eating disorder behaviors in a naturalistic context.

In this work, greater ecological validity was sought by considering appearance comparisons that occurred naturally rather than comparisons produced in an experimental context. However, the reported appearance comparisons and the obtained indicators were not collected immediately after the comparisons were made, so memory errors may have occurred. Other studies may ask participants to record comparisons as soon as they are made, allowing for greater ecological validity.

Furthermore, to prevent experimental death and demotivation of the participants, a single data collection was used, without resorting to Ecological Momentary Assesment (EMA), asking for only one appearance comparison. Despite this methodological difference, there still were found similar results to studies that used EMA [7,8,9,11,15]. However, according to Arigo et al. [13], analyzing only one comparison from each participant can remove the significant variation that occurs in comparisons in real contexts and also the temporal sequence regarding predictors and results.

The single data collection also influenced the number of responses received, resulting in few reported downward and lateral appearance comparisons, which may have contributed to the fact that some of the expected results were not found. It is recommended that future investigations use EMA, to examine appearance comparisons in their natural context, allowing the generalization of results to real life [9]. A larger number of responses should also be gathered. To achieve this goal, Fardouly et al. [8] suggest that all comparisons made throughout the day be requested or that data collection last longer.

Another recommendation by Fardouly et al. [8] that seems appropriate is to allow participants to report more than one appearance comparison at the same time, as several comparisons may be made, considering different physical attributes. In addition, it would be important to ask participants about other types of comparison besides appearance, such as performance comparisons, because there may be an interaction between the different types of comparisons made.

Data generalizability is also limited to the studied population—Portuguese university students, female gender, and aged between 17 and 25 years. Despite that, young women have higher levels of association between appearance comparisons and body dissatisfaction [5], thus being an appropriate population for the study of appearance comparisons. However, future studies should include a larger and more diverse sample in terms of gender, age, and ethnicity, among others, to investigate the phenomenon of appearance comparisons on a broader scale.

The analyzes of this study were cross-sectional; therefore, it is not possible to affirm the direction of the association between the measures related to appearance comparisons and body dissatisfaction, positive affect, negative affect, and eating pathology, nor to study the causality between these variables.

Finally, some constructs that may be associated with appearance comparisons, body dissatisfaction, positive affect, negative affect, and eating pathology, such as self-esteem, perfectionism, and the internalization of appearance ideals, were not evaluated. The literature supports the relevance of these other variables. Succinctly, Taniguchi and Ebesu Hubbard [4] found that self-esteem was more strongly associated with body dissatisfaction than appearance comparisons. Vartanian and Dey [12] encountered that the idealization of the thin ideal mediates the association between appearance comparison tendency and body dissatisfaction. Additionally, McComb and Mills [42] found that high levels of perfectionism associated with physical appearance were predictors of lower levels of satisfaction with weight after appearance comparisons to models. The inclusion of these and other variables could bring greater validity, control, and richness to future studies.

### 4.2. Implications

Festinger [2] suggested that the greater the relevance of the ability to the group, the greater the pressure for uniformity in relation to this ability. Assuredly, appearance is given great importance in today’s society, and there is an elevated pressure to conform to appearance norms, especially regarding women. According to Strahan et al. [31], appearance norms tend to be more explicit and homogeneous for women. Furthermore, women demonstrate a self-deprecating evaluation and comparison pattern when describing their appearance and weight. We can conclude with a normative dissatisfaction with weight and body shape in society and a pervasiveness of the negative effects of appearance comparisons on women [9].

Appearance comparisons are associated with body dissatisfaction, negative affect, and eating pathology. According to Betz et al. [43], a focus on reducing these comparisons can be productive for social change. In this way, it is important to increase awareness about this type of comparison and its implications. Moreover, if these comparisons induce psychological distress and maladaptive behaviors, it is necessary to guide the clinical work to mitigate these consequences. In light of this, Fairburn [44] addresses appearance comparisons in his eating disorder treatment protocol by educating clients about how unfavorable comparisons with others constitute a form of weight and body shape verification that maintains a pathological preoccupation with weight and body shape. Cash and Grant [45] developed a cognitive–behavioral body image treatment program in which they address the identification and challenge of cognitive errors associated with appearance comparisons. Furthermore, Schaefer and Thompson [46] recommend that in case their clients exhibit high levels of appearance comparisons, clinicians should provide psychoeducation about the harmful effects of these comparisons, introduce comparison monitoring, and teach specific strategies to challenge appearance comparison tendencies.

Promoting body positivity and/or neutrality may also serve to counteract possible adverse effects of appearance comparisons. According to Halliwell [47], body positivity involves the rejection of appearance ideals, which may influence the choice of target, attribute, and direction of comparison, making comparisons less threatening. Additionally, Andrew et al. [48] found that body appreciation was negatively associated with social comparisons. Body positivity and neutrality can be adopted by valuing body functionality over body aesthetics [49] and also by body flexibility, that is, the ability to experience negative thoughts or emotions about the body without trying to avoid or change them. Indeed, Tan et al. [50] found that, when experiencing body dissatisfaction, women with greater body image flexibility are less likely to engage in appearance comparisons than women with less body image flexibility.

Body positivity and neutrality can also be encouraged by appreciating more diverse bodies. The presentation of different shapes and sizes and women of different ages, ethnic groups, abilities, and sexualities may shape women’s body attitudes in a more positive way [43]. Moreover, McKee et al. [51] state that having more demographically diverse friendships can protect women from the negative consequences associated with appearance comparisons.

## 5. Conclusions

There is extensive literature on the characteristics of appearance comparisons and their association with psychological indicators and well-being. In this work, some of these characteristics and associations were examined in a sample of Portuguese university students. Among this sample, 98.71% reported making appearance comparisons, and 42.15% of these participants referred to making them frequently or always. Higher reported frequencies of appearance comparisons were associated with higher levels of body dissatisfaction, negative affect, and eating pathology. Furthermore, appearance comparisons with acquaintances were the most frequent, followed by models/celebrities, strangers, and close peers. Reported mediums of appearance comparison (in person or through media) had similar proportions, and there was a greater proportion of upward appearance comparisons in comparisons made through media than in person. There was a greater number of upward appearance comparisons than laterals and downward appearance comparisons. Additionally, upward appearance comparisons were linked to greater negative affect and eating pathology than lateral appearance comparisons and higher body dissatisfaction than lateral and downward appearance comparisons. BMI, target, and medium were not moderators of the association between the direction of the appearance comparison and body dissatisfaction, negative affect, and eating pathology. Finally, higher body dissatisfaction was found in upward appearance comparisons to close peers as opposed to upward appearance comparisons to models/celebrities. Perceived attainability of the target’s appearance did not mediate this association and did not vary significantly between target levels. The results of the present study provide useful information for understanding appearance comparisons among female university students and their potential implications in clinical and community domains.

## Figures and Tables

**Table 1:** Observed Means and Standard Errors of Body Dissatisfaction, Negative Affect, Eating Pathology, and EDE-Q Subscales Within Levels of Reported Frequency of Appearance Comparisons.

Dependent Variables	Rarely M (SE)	Occasionally M (SE)	Frequently M (SE)	Always M (SE)
Body Dissatisfaction	1.77 (0.10) ^O, F, A^	2.32 (0.07) ^R, F, A^	3.06 (0.07) ^R, O, A^	4.00 (0.14) ^R, O, F^
Negative Affect	12.33 (0.86) ^F, A^	14.20 (0.59) ^F, A^	21.21 (0.64) ^R, O, A^	25.26 (1.25) ^R, O, F^
Eating Pathology	0.58 (0.14) ^F, A^	0.93 (0.10) ^F, A^	2.13 (0.11) ^R, O, A^	3.26 (0.20) ^R, O, F^
Restraint	0.50 (0.17) ^F, A^	0.70 (0.12) ^F, A^	1.51 (0.13) ^R, O, A^	4.23 (0.10) ^R, O, F^
Eating Concern	0.28 (0.13) ^F, A^	0.36 (0.09) ^F, A^	1.36 (0.10) ^R, O, A^	2.06 (0.19) ^R, O, F^
Weight Concern	0.77 (0.17) ^F, A^	1.20 (0.12) ^F, A^	2.65 (0.13) ^R, O, A^	3.87 (0.25) ^R, O, F^
Shape Concern	0.79 (0.16) ^O, F, A^	1.45 (0.11) ^R, F, A^	3.00 (0.12) ^R, O, A^	4.58 (0.23) ^R, O, F^

Note: Significant (*p* ≤ 0.05) differences of observed means of the quantitative variables between each reported frequency are indicated by superscripts (^R, O, F, A^), regarding the reported frequencies Rarely, Occasionally, Frequently, and Always, respectively.

**Table 2:** Observed Means and Standard Errors of Body Dissatisfaction, Negative Affect, Eating Pathology, and EDE-Q Subscales Within Levels of Direction of Appearance Comparison.

Dependent Variables	Upward M (SE)	Lateral M (SE)	Downward M (SE)
Body Dissatisfaction	2.74 (0.56) ^L, D^	1.76 (0.17) ^U^	1.85 (0.26) ^U^
Negative Affect	17.93 (0.47) ^L^	12.07 (1.41) ^U^	12.58 (2.19)
Eating Pathology	1.55 (0.08) ^L^	0.75 (0.25) ^U^	1.30 (0.38)
Weight Concern	1.95 (0.10) ^L^	0.93 (0.30) ^U^	1.62 (0.46)
Shape Concern	2.26 (0.10) ^L^	1.03 (0.30) ^U^	1.62 (0.47)

Note: Significant (*p* ≤ 0.05) differences of observed means of the quantitative variables between each direction of comparison are indicated by superscripts (^U, L, D^), regarding the directions Upward, Lateral, and Downward, respectively.

**Table 3:** Observed Means and Standard Errors of Body Dissatisfaction Within Levels of Target in Upward Appearance Comparisons.

Dependent Variables	Close Peer M (SE)	Acquaintance M (SE)	Stranger M (SE)	Model/Celebrity M (SE)
Body Dissatisfaction	3.05 (0.15) ^M^	2.88 (0.10)	2.67 (0.12)	2.51 (0.10) ^C^

Note: Significant (*p* ≤ 0.05) differences of observed means of the quantitative variables between each target of comparison are indicated by superscripts (^C, M^), regarding the targets Close Peer and Model/Celebrity, respectively.

## Data Availability

The data are not available due to the obtained consent being restrained to this specific study.
